# The Nitric Oxide Production in the Moss *Physcomitrella patens* Is Mediated by Nitrate Reductase

**DOI:** 10.1371/journal.pone.0119400

**Published:** 2015-03-05

**Authors:** Rigoberto Medina-Andrés, Alejandro Solano-Peralta, Juan Pablo Saucedo-Vázquez, Selene Napsucialy-Mendivil, Jaime Arturo Pimentel-Cabrera, Martha Elena Sosa-Torres, Joseph G. Dubrovsky, Verónica Lira-Ruan

**Affiliations:** 1 Laboratorio de Fisiología y Desarrollo Vegetal, Centro de Investigación en Dinámica Celular, Instituto de Investigación en Ciencias Básicas y Aplicadas Universidad Autónoma del Estado de Morelos, Cuernavaca, Morelos, México; 2 Departamento de Química Inorgánica y Nuclear, Facultad de Química, Universidad Nacional Autónoma de México, México D.F., México; 3 Departamento de Biología Molecular de Plantas, Instituto de Biotecnología, Universidad Nacional Autónoma de México, Cuernavaca, Morelos, México; 4 Laboratorio Nacional de Microscopia Avanzada, Universidad Nacional Autónoma de México, Cuernavaca, Morelos, México; Universidade de São Paulo, BRAZIL

## Abstract

During the last 20 years multiple roles of the nitric oxide gas (•NO) have been uncovered in plant growth, development and many physiological processes. In seed plants the enzymatic synthesis of •NO is mediated by a nitric oxide synthase (NOS)-like activity performed by a still unknown enzyme(s) and nitrate reductase (NR). In green algae the •NO production has been linked only to NR activity, although a *NOS* gene was reported for *Ostreococcus tauri* and *O. lucimarinus*, no other Viridiplantae species has such gene. As there is no information about •NO synthesis neither for non-vascular plants nor for non-seed vascular plants, the interesting question regarding the evolution of the enzymatic •NO production systems during land plant natural history remains open. To address this issue the endogenous •NO production by protonema was demonstrated using Electron Paramagnetic Resonance (EPR). The •NO signal was almost eliminated in plants treated with sodium tungstate, which also reduced the NR activity, demonstrating that in *P. patens* NR activity is the main source for •NO production. The analysis with confocal laser scanning microscopy (CLSM) confirmed endogenous NO production and showed that •NO signal is accumulated in the cytoplasm of protonema cells. The results presented here show for the first time the •NO production in a non-vascular plant and demonstrate that the NR-dependent enzymatic synthesis of •NO is common for embryophytes and green algae.

## Introduction

The multiple roles of nitric oxide (•NO) in plant physiology have been extensively studied for almost two decades [1–3]. Nowadays, it is recognised that •NO is a fundamental signalling molecule during plant development, from germination to floral set and senescence [4–6] as well as in the response to biotic and abiotic stress conditions [7–9]. Besides the study of •NO functions, another appealing research topic is the analysis of the mechanisms to generate •NO in plant systems. In land plants two main enzymatic systems involved in •NO production are recognized, a nitric oxide synthase (NOS-like) activity and •NO production by nitrate reductase (NR, EC 1.7.1.1–3) [10,11]. In mammals the NOS enzyme oxidizes L-arginine to •NO and citrulline [12]. Such activity has been found in a variety of plant organs such as *Lupinus albus* roots and nodules [13], *Zea mays* root tips [14] and in *Nicotiana tabacum* epidermal cells and cell cultures [15,16]. The decrease in •NO content after the application of animal NOS inhibitors reinforced the idea that plants has NOS enzyme [17–19]. However, neither a NOS protein nor a gene has been found in embyophytes [10, 20]. In the green algae, *Ostreococcus tauri* and *O. lucimarinus*, a functional NOS protein was reported, but no other algae or land plant sequence matches with the *O. tauri NOS* gene [21], leaving the identity of a plant NOS unresolved. In addition to the classical role of NR in reducing nitrate to nitrite, this enzyme reduces the nitrite to •NO in different plant species [22–25]. The *Arabidopsis thaliana* mutant *nia1/nia2* that lacks NR produces less •NO and is more susceptible to bacterial and fungal infections [26–29] demonstrating the importance of NR-derived •NO in plant physiology. In green algae, the synthesis of •NO has been reported in *Scenedesmus obliquus*, *Chlamydomonas reinhardtii* and *Chlorella sorokiniana* [30–32]; the •NO production in *S. obliquus* and *C. sorokiniana* was nitrite-dependent and insensitive to NOS inhibitors. According to this, the compelling question of the evolution of enzymatic •NO sources in land plants emerges.

In land plants, all the studies regarding •NO synthesis have been performed in angiosperms and gymnosperms [19,33,34] leaving a gap of information concerning the •NO synthesis in the rest of the embryophytes (i.e. bryophytes and pteridophytes). In evolutionary terms, the bryophytes are widely accepted as the modern representatives of early land plants [35,36]. Thus the study of the enzymatic synthesis of •NO in the basal land plants will provide valuable information about the enzymatic synthesis of •NO in the Plant kingdom. The moss *Physcomitrella patens* has been used as a model system for comparative analysis between basal and higher land plants [37–40]. It has been reported that *P. patens* possesses three *NIA* genes [41] as well as the NR activity [42]. Thus, the aim of this work was to evaluate whether *P. patens* is capable of •NO production and to establish the possible role of NR in this process.

Measuring •NO in live plants is a challenge that has promoted the development of direct and indirect techniques to detect this molecule, recently reviewed in [43]. Among them, the Electron Paramagnetic Resonance (EPR) technique stands out because it accurately detects •NO in crude extracts using a specific “spin-trap” [44]. This technique has been used to detect •NO in *Pisum sativum* leaves [45], *Glycine max* chloroplasts [46] and to characterize the •NO production in the *Arabidopsis* mutant *nia1/2/noa1* [47]. Other methods, such as epi-fluorescence and confocal laser scanning microscopy (CLSM) using fluorescence probes are common in •NO research. From the family of diaminofluoresceins (DAF), 4,5-diaminofluorescein diacetate (DAF-2DA) is preferred because it enters the cell and reacts with •NO to produce the fluorescent triazole DAF-2T [48] allowing the identification and localization of •NO inside cells. This technique has been used to monitor •NO production in green algae [31], gymnosperms [49], and angiosperms [45, 50].

Here, the presence of •NO in *P. patens* protonema was detected using EPR spectroscopy and CLMS. As no mutants of *NIA* genes are available, a pharmacological strategy to inactivate NR using sodium tungstate was successfully applied. Plants with reduced NR activity had a •NO production drastically reduced, indicating that in the moss *P. patens* protonema, NR is the main source for •NO production.

## Materials and Methods

### Plant growth conditions and tungstate treatments

Explants from *P. patens* (Hewd) B.S.G protonema were cultivated on solid Knop medium (0.8% Agar) [51] covered with sterile cellophane discs. Plants were cultivated in a growth chamber at 21°C with 16/8h light/dark period. Light intensity was 30 μmol m^-2^ s^-1^.

To analyse the effect of sodium tungstate on *P. patens* protonema growth, seven day-old protonema pieces were transferred from simple Knop medium to the same medium supplemented with different concentrations of sodium tungstate (Sigma-Aldrich). The plants were grown for seven days under treatment. At the day of the transfer (day 0) and seven days later when the experiment ended (day 7) the Petri dishes with the protonema cultures were photographed with a Nikon Colpix5000 camera. To analyse the effect of high nitrate concentration (8.4 mM Ca(NO_3_)_2_) on *P. patens* growth, the same assay was performed adding nitrate to Knop media alone and to Knop media supplemented with 30 μM sodium tungstate. The area covered by each plant at day zero and day seven were measured using ImageJ software (http://rsb.info.nih.gov/ij/). The relative growth rate was calculated using the following formula: (*ln a*_*f*_ *—ln a*_*0*_)*t* ^*-1*^ [52] where *a*_*f*_ and *a*_*0*_ *are* the areas occupied by the plant at final day of growth (day 7) and at the beginning of the experiment (day 0) and *t* is the duration of growth equal to 7 days.

### Nitrate reductase activity

Nitrate reductase activity was measured following an established method [53]. The protonema was ground with four volumes (v/w) of cold extraction buffer (250 mM Tris-HCl, pH 8.0, 1 mM EDTA, 1 μM Na_2_MoO_4_, 5 μM FAD, 3 mM DTT, 1% BSA, 12 mM β-mercaptoethanol and 250 μM PMSF). One volume of pre-warmed (25°C) assay buffer (40 mM KNO_3_, 8 mM Na_2_HPO_4_, 20 mM NaH_2_PO_4_ [pH7.5] and 0.2 mM NADH) was added and the mixture was incubated at 25°C. After 0, 5, 10 and 15 min, 100μl aliquots were removed from the assay mixture and the reaction was stopped by adding 25 μl of 0.6 M zinc acetate, the samples were kept for 20 min at room temperature, then 100 μl of 1% sulphanilamide dissolved in 3 N HCl supplemented with 100 μl 0.02% N-(1-naphthyl)-ethylenediamine were added. The mixture was incubated for 20 min at room temperature, centrifuged at 18,000 x g and the nitrite was measured by spectroscopy at 540 nm. Nitrite concentration expressed in μmol h^-1^ g^-1^ FW was calculated based on a nitrite standard curve. The nitrite content of control and tungstate treated plants was obtained from the samples taken at time 0. Total soluble proteins were obtained by grinding 0.1 g of protonema in 400 μl of the extraction buffer (250 mM Tris-HCl [pH 8.0], 250 μM PMSF, 12 mM ß-mercaptoethanol, 1 mM EDTA). The resulting solution was cleared by centrifugation at 13 000 x g for 5 min. Concentration of soluble proteins was quantified using Bradford reagent binding assay (Sigma-Aldrich) using bovine serum albumin as standard.

### •NO detection by EPR

Plant extracts were obtained by homogenizing 2.0 g of protonema explants with 500 μl of 0.1 M phosphate buffer (pH 7.2), previously flushed with argon, then centrifuged at 13,000 x g for 10 min. The supernatants (750μl) were mixed with an equal volume of freshly made 20 mM degassed citrate buffer containing 30 mM N-methyl-D-glucamine dithiocarbamate (MGD) (Santa Cruz) and 1 mM FeSO_4_-7H_2_O in deionized water [54, 55]. Then the mixtures were incubated for 1h at room temperature to allow the formation of the (MGD)_2_Fe(II)NO complex. Samples were maintained on ice and immediately measured. Since •NO reacts rapidly with O_2_ the sample preparation was performed in an anoxic chamber. EPR spectra were measured under non-saturating conditions of microwave power on a Bruker Elexys E500 X-band spectrometer (in a quartz flat cell at 297K; microwave power, 20.0 mW; modulation frequency, 100KHz; modulation amplitude, 3G; time constant, 20.48 ms). The spectra were simulated using the Bruker software, and *g*-values were calculated by measuring the magnetic field and the microwave frequency.

### •NO detection by CLMS

Protonema samples (grown as indicated before) from simple media (control) and media with 30 μM sodium tungstate were incubated for 15 min in the dark in 0.1 M phosphate buffer (pH 7.4) containing 20 μM of 4,5-diaminofluorescein diacetate (DAF-2DA) (Calbiochem). Plants treated with the •NO scavenger, 2-(4-carboxyphenyl)-4,4,5,5-tetramethylimidazoline-1-oxyl-3-oxide (cPTIO) (Sigma-Aldrich), were pre-incubated for 45 min with 200 μM cPTIO in 0.1 M phosphate buffer (pH 7.4). Afterwards, the samples were incubated for 15 min in the same buffer supplemented with 20 μM DAF-2DA and 200 μM cPTIO. Then the plants were mounted on slides in the same solution and analysed under the microscope. All images were collected with a confocal Zeiss LSM510 inverted microscope equipped with 40x Plan Neofluar NA 0.75 objective lens. DAF-2DA fluorescence was excited with the 488 nm line of Argon laser (with HFT UV/488/543/633 DM, BP 500–530 nm settings). Images were collected using the same specific set of parameters for laser power, photomultiplier gain level, pixels (0.2197 microns) and pinhole size. The obtained data corresponded to a stack of images of 1024 x 1024 pixels, each one composed of two channels (fluorescence and bright field).

A quantitative method to compare the fluorescence intensity associated with •NO production in *P. patens* tissue was developed. From each single image, the information from bright field and fluorescence channels were combined to identify the pixels corresponding to the regions of interest (ROI) associated with fluorescent signal; this process eliminated the pixels of the background of the image. To analyze the set of pixels on the ROI’s, we have developed an algorithm to calculate the fluorescence intensity histogram and to normalize its intensity distribution relative to its own area.

Applying the algorithm to the set of images associated with each experimental treatment, a set of histograms of gray intensity levels was obtained from where an average curve that describes the probability distribution of the gray intensity levels associated to the specific experimental treatment was obtained. The autofluorescence associated to the sample without DAF-2DA was calculated and subtracted from these curves. The statistical independence between the resultant distributions was determined using a Kolmogorov-Smirnov test. Finally, integrating the intensity distribution for every treatment, we obtained the total fluorescence corresponding to the different experimental conditions. The highest fluorescence corresponding to the control DAF-2DA sample was used to normalize the total intensity associated to all the experimental conditions. Two biological replicates were performed (n = 22–28 for each treatment)

## Results

### Nitrate reductase activity inhibition

As the interest of this work is to examine whether *P. patens* produces •NO in a NR-dependent manner and in light of the lack of null *nia* mutants, a pharmacological approach to reduce NR activity was employed. It is believed that tungsten ion substitutes for a molybdenum ion during the synthesis of the molybdate cofactor, and the addition of tungsten to growth media successfully inactivated NR in tobacco and rice leaves [45,56]. Thus, *P. patens* protonema were cultivated in simple Knop medium or in medium supplemented with increasing concentrations of sodium tungstate.

**Fig 1:**
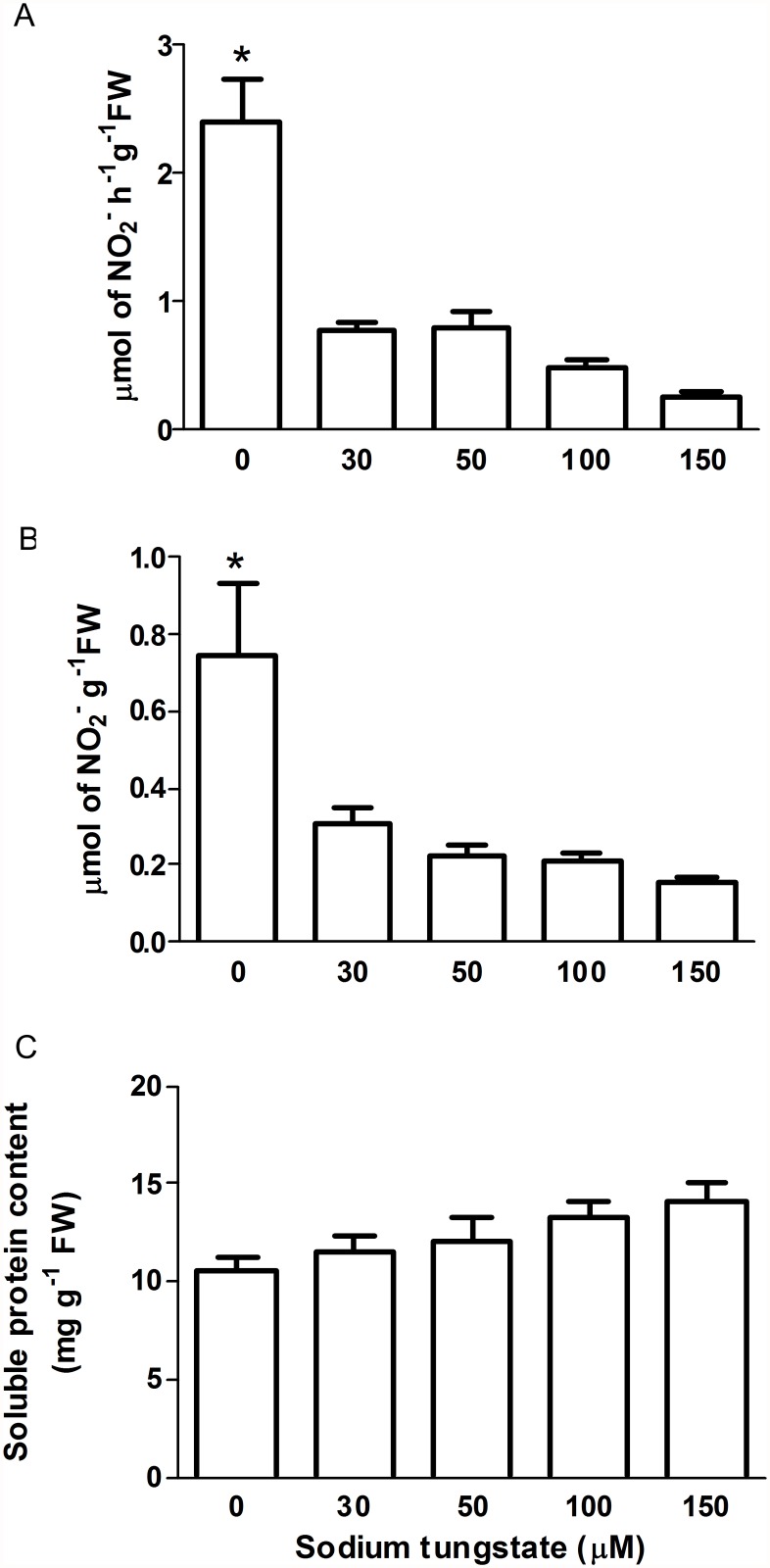
Analysis of the effect of sodium tungstate in *P. patens* protonema. A) Nitrate Reductase activity. B) Plant endogenous nitrite content. C) Plant total soluble proteins. Plants grew for seven days in simple Knop medium or media supplemented with increasing concentrations of sodium tungstate. Data are mean of three independent experiments. Data were analyzed by one-way ANOVA and Tukey´s multiple comparison test (*n* = 9 in panel A, *n* = 3 in panels B and C). Asterisk denotes statistically significant difference (*P* < 0.05). Error bars denote SE.

As shown in (Fig. 1A) NR activity in the untreated (control) plants was 2.4 μmol h^-1^ g^-1^ FW, which is comparable to the range of NR activity reported for other plants as *A. thaliana*, *Nicotiana plumbaginifolia* and *Solanum lycopersicum* leaves (15, 1.9 and 0.38 μmol h^-1^ g^-1^ FW, respectively) [57–59].

**Fig 2:**
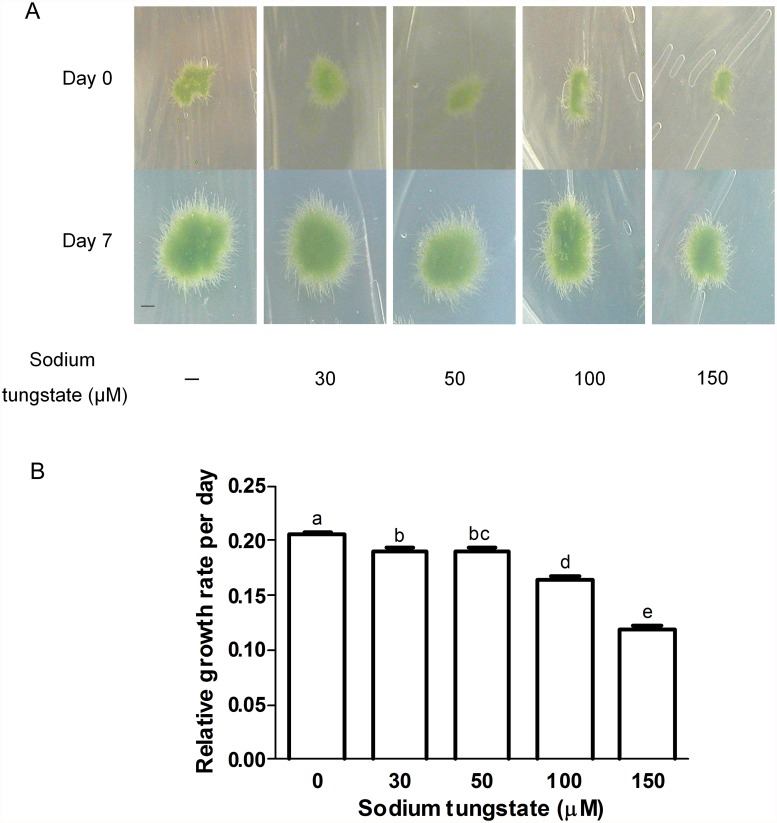
Effect of sodium tungstate on *P. patens* protonema growth. A) Plants grew for seven days on simple Knop medium or on media supplemented with the indicated concentrations of sodium tungstate. Pictures were taken at the beginning and end of the experiment, Day 0 and Day 7, respectively. Scale bar = 3 mm B) Plant relative growth rate was measured according to 52. (*ln a*_*f*_ *—ln a*_*0*_ *)t* ^*-1*^ where *a*_*f*_ and *a*_*0*_ are the plant area at day 7 and at day 0, respectively. *t* is the time growth in days = 7. Data are mean of three independent experiments. Data were analyzed by one-way ANOVA and Tukey´s multiple comparison test (*n* = 84). Different letters indicate a statistically significant difference (*P* < 0.05). Error bars denote SE.

As expected, sodium tungstate decreased the NR activity; plants growing with 30 μM sodium tungstate had only 32% of the NR activity from untreated control plants. Moreover, the activity was reduced to 10% of that from control plants during 150 μM sodium tungstate treatment. To confirm the NR inactivation the nitrite content from control and treated plants was measured (Fig. 1B). Nitrite is the product of NR activity, thus when the enzyme is inactive less product should be formed. Indeed, plants treated with 30 μM and 150 μM sodium tungstate had 41% and 21% of the nitrite content of control plants, respectively. These results demonstrated that NR activity was effectively blocked by the tungstate present in the growth medium. To analyse the possible effect of reduced NR activity on nitrogen metabolism the total soluble protein content from control and tungstate-treated protonema was measured. As shown in Fig. 1C, the total protein level did not change between control and treated plants, suggesting that the NR activity still present in plants treated with sodium tungstate was enough to maintain the tissue protein demands. To select the most suitable tungstate concentration for evaluating the NR role in •NO synthesis, the growth of control and treated plants was compared (Fig. 2). Tungstate treatments lasted for seven days and the relative growth was calculated from the pictures at the beginning and end of the experiment (Fig. 2A). The statistical analysis showed that tungstate treatment reduced the relative plant growth rate in a dose-dependent manner (Fig. 2B). The most severe effect was at 150 μM which reduced the growth rate to 58% of that in the control. As 30 μM sodium tungstate had a subtle effect on the relative plant growth rate but the enzyme lost 68% of its activity (Fig. 1A), this concentration was used in the treatments to analyse the NR contribution to •NO synthesis.

### Detection of •NO in P. patens protonema by EPR and CLMS

**Fig 3:**
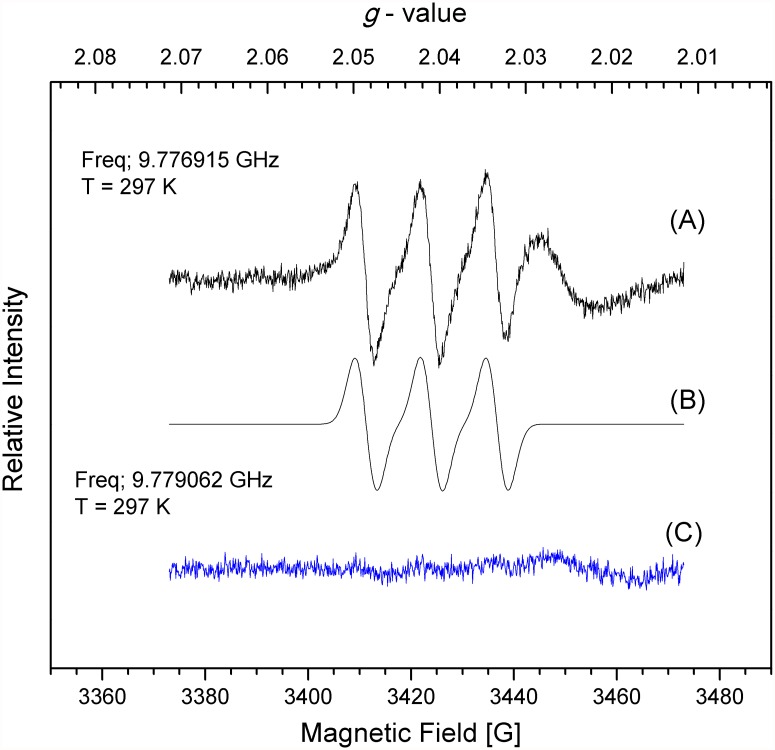
Nitric oxide detection by EPR in P. patens protonema. A) Plants grew for seven days on simple Knop media or C) on medium supplemented with 30 μM sodium tungstate. Homogenized tissues were mixed with (MGD)_2_Fe(II) and incubated for 1h. EPR spectra were recorded at approximately 9.8 GHz (X-band) and 100 kHz modulation (Microwave frequency, 9.776915 GHz for A and 9.779062 GHz for C; microwave power, 2.0 mW; modulation amplitude: 0.3 mT; T, 297 K). Each graph is a representative spectrum of four independent experiments. B represents the simulated spectrum of A.

The multiple roles of •NO in plant physiology have been shown in green algae and in higher plants [60, 61], but its production in non-vascular plants has not been demonstrated. Thus, the endogenous •NO in *P. patens* was detected using EPR and the spin trap (MGD)_2_Fe(II). The EPR spectrum of (MGD)_2_Fe(II)NO adduct at 297 K (Fig. 3) isolated from the control protonema extracts revealed a well-resolved three-line spectrum with an isotropic g value = 2.040 and an hyperfine coupling, a^N^, value of 13 G typical of the EPR signal of (MGD)_2_Fe(II)NO compounds in aqueous solution [54] demonstrating that *P. patens* produce •NO during normal protonemal growth.

**Fig 4:**
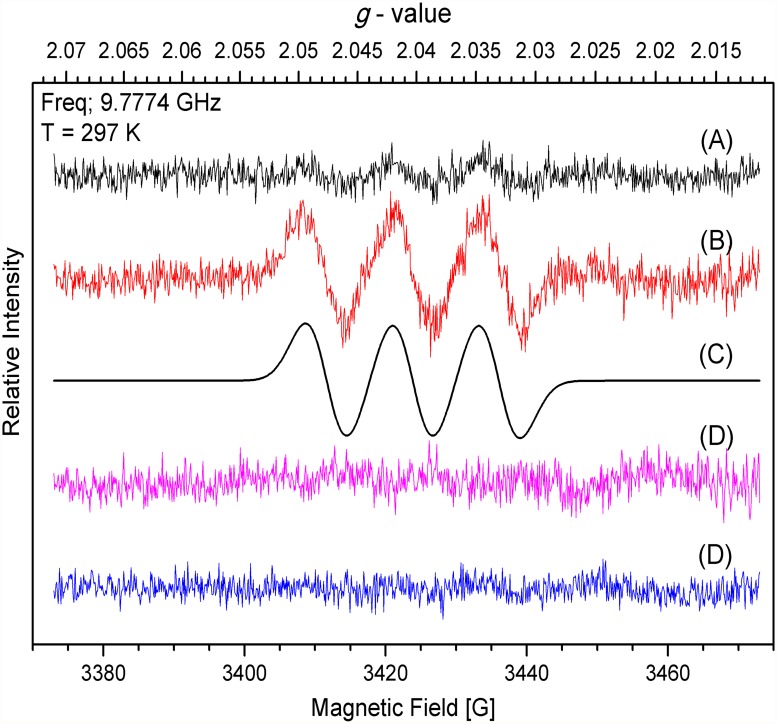
Nitric oxide detection by EPR in P. patens protonema growing in high nitrate. The EPR assay was performed as in Fig. 3 using plants that grew for seven days on A) Knop media, B) media added with 8.4 mM Ca(NO_3_)_2_, D) Knop media added with 30μM sodium tungstate and E) media added with 8.4 mM Ca(NO_3_)_2_ and 30μM sodium tungstate. EPR spectra were recorded at approximately 9.8 GHz (X-band) and 100 kHz modulation (Microwave frequency, 9.777400 GHz for B) and microwave power, 2.0 mW; modulation amplitude: 0.3 mT; T, 297 K). Each graph is a representative spectrum of four independent experiments. C) represents the simulated spectrum of B).

Under the same concentration of extract and spin trap, the •NO signal decreased almost completely in plants that grew on 30 μM sodium tungstate indicating that the •NO production in *P. patens* protonema was dependent on NR activity. To strength this conclusion the •NO production was analysed in protonema that grew in Knop media supplemented with high nitrate concentration 8.4 mM Ca(NO_3_)_2_ (Knop media contains 4.2 mM Ca(NO_3_)_2_ [Fig. 4A]). As expected the •NO amount increased (Fig. 4B) compared to standard nitrate concentration. The •NO signal was almost lost in plants grown in standard media and media with high nitrate both supplemented with sodium tungstate (Fig. 4D and E) confirming that NR is the enzyme responsible for the •NO production. The plant growth rate and NR activity in plants growing in high nitrate did not change compared with plants growing in simple Knop media (S1 Fig.)

**Fig 5:**
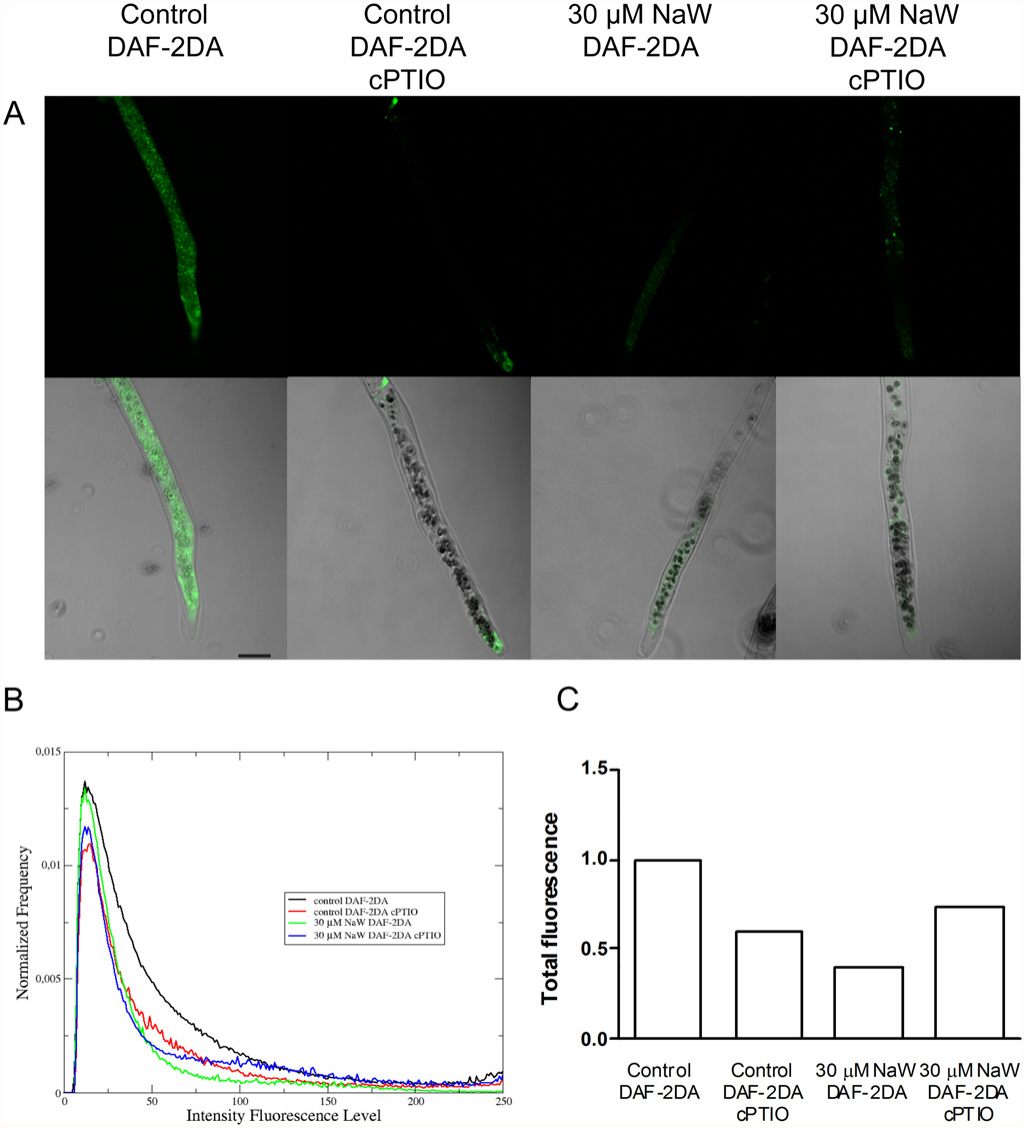
Nitric oxide detection in *P. patens* protonemal cells by confocal laser scanning microscopy. A) DAF-2DA green emission by protonemal cells grown for seven days on Knop medium or medium supplemented with 30 μM sodium tungstate. Protonema was incubated in growth medium supplemented with 20 μM DAF-2DA for 15 min. Some plants were preincubated with 200 μM cPTIO for 45 minutes and then with DAF-2DA. Green signal indicates •NO production. The micrographs show pairs of representative laser confocal microscopy and bright-field images of the protonemal cells. Scale bar = 50 μm. B) Normalized average histograms, each curve describes the DAF-2DA green emission intensity distribution associated to a specific experimental treatment. C) For each treatment, the total DAF-2DA green emission intensity was obtained based on the area under the curves shown in B, and normalized according to DAF-2DA green signal intensity from untreated control. Data from two independent experiments (n = 22–28). The statistical independence between the distributions (B) was calculated using the Kolmogorov-Smirnov test.

To further characterize the •NO production in *P. patens* protonema an analysis of •NO localisation was performed using the DAF-2DA fluorescent probe and CLSM (Fig. 5A). The •NO signal was detected in the cytoplasm of control plants, confirming the presence of •NO in protonema. The fluorescence signal was significantly decreased in protonema of control plants incubated with cPTIO as well as in plants treated with 30 μM of sodium tungstate with or without cPTIO. The quantitative analysis of the DAF-2DA emission assay presented in Fig. 5B showed that the •NO signal from control plants treated with 200 μM cPTIO was 42% of that in the control plants, indicating that the DAF-2DA green emission in untreated control was due to •NO present in cells (Fig. 5C). Moreover, the DAF-2DA signal in protonema of plants treated with sodium tungstate was reduced to the same level as that in control plants treated with cPTIO, supporting the observation that •NO is produced mainly by NR activity. The addition of cPTIO to tungstate-treated plants did not change the •NO signal intensity, confirming that in these plants the •NO synthesis or accumulation was blocked.

## Discussion

The •NO synthesis mediated by NR is present in green algae and seed plants. In the latter, the NOS-like activity has been described in several species, but the protein(s) that perform such activity remains unknown. Interestingly, there is little information about •NO synthesis and function in basal land plants. Recently we described a family of three *NIA* genes in *P. patens* [41]. Here, the activity of the enzymes encoded by those genes was verified and their role in •NO synthesis examined. The NR activity of protonema growing in normal conditions (Fig. 1A) is comparable to NR from different plant species [57– 59]. However, the result obtained here is different from a report in which the NR activity from *P. patens* is 0.24 μmol h^-1^ g^-1^ FW [42]. The age and developmental stage of the plants used in that work were not reported. Here, the NR activity corresponds to two week-old plants formed by growing protonema tissue, and thus it is likely that the nutrient demands in this developmental stage could explain the greater NR activity found in our study. The effectiveness of sodium tungstate treatment to reduce NR activity in *P. patens* provided an appropriate system to evaluate the role of NR in •NO synthesis in this plant, as an alternative to the use of *nia* mutants that are not available. The inactivation with 30 μM sodium tungstate was enough to reduce NR activity by 68% without affecting total protein content (Fig. 1C) and a decrease of only 8% on relative growth rate (Fig. 2B). The suitability of the use of tungstate to inactivate NR in studies regarding •NO synthesis has been questioned because in plants the tungsten ion substitutes the molybdenum ion in three more enzymes besides NR: sulphite oxidase, xanthine dehydrogenase and aldehyde oxidase. The inactivation of such enzymes could alter the cell metabolism and indirectly the •NO synthesis [62]. Only xanthine dehydrogenase has been linked to •NO production in mammals [62, 63] but the recombinant protein from plants is unable to support •NO synthesis [64]. On the other hand, it has been shown in *C. sorokiniana nia* mutant that treatment with 100 μM tungstate did not alter the •NO emission, detected by gas phase chemiluminescence, indicating that the potential inhibition of the other Mo enzymes do not participate in the synthesis of •NO [32]. The protein content in control and treated plants did not change (Fig. 1C) indicating that the nitrogen metabolism was not affected by the sodium tungstate treatment. Moreover, the tungstate concentration used here (30 μM) was lower than the concentration used by other authors that ranges from 1 to 0.1 mM (reviewed by [62]) ruling out the possible stress effect of tungstate in *P. patens*. The inactivation of NR by sodium tungstate treatment on *P. patens* protonema resulted in a phenotype similar to that obtained in other plant models with reduced NR activity. The *A. thaliana* single mutant *nia1*, has a NR activity of only 10% of that of wild type and presents normal shoot growth, whereas the double mutant *nia1/nia2* had NR activity that is only 0.05% of that of the wild type and the plant size is severely reduced [65]. In the case of *P. patens*, plants treated with 30 μM sodium tungstate had a NR activity representing 32% of that in control plants, a normal total protein level and almost normal growth. Accordingly, it was concluded that the best conditions to analyse the possible role of NR in •NO accumulation in *P. patens* was the treatment with 30μM sodium tungstate.

As discussed before, EPR is an excellent method to detect •NO that has been used successfully in several plants [45–47]. Here, the g value at 2.040 and a hyperfine coupling of 13 G obtained by the EPR technique was used to demonstrate beyond any doubt the existence of endogenous •NO in *P. patens* protonema. The results presented in Figs. 3 and 4 show for the first time that non-vascular plants produced •NO during normal growth, and that the •NO signal from plants with low NR activity is dramatically reduced demonstrating that NR is the main source of •NO in *P. patens*. The •NO production by NR has been demonstrated in different organs of a variety of plant species. Rockel *et al*., [24] established the nitrate dependent •NO production in sunflower and spinach leaves. The constitutive expression of *NIA1* gene from *Eucalyptus grandis* in *A. thaliana* resulted in increased •NO levels in transgenic plants [66]. Moreover, the *A. thaliana* double mutant *nia1/nia2* produce less •NO and is more susceptible to bacterial infections [26, 27]. These results demonstrate the importance of NR in •NO synthesis in higher plants. Also in green algae the NR-dependent •NO production was proved in *C. reinhardtii* and *S. obliquus* [30, 31] and it is probably present in other species. The enhanced •NO production observed in plants growing in high nitrate (Fig. 4) indicates that *P. patens* NR, as the other NR from plants, increases the •NO production when more substrate is available.

A commonly used technique to analyse •NO location in plant cells and tissues is epi-fluorescence or CLMS with DAF-2DA used as a probe [18, 62, 63]. Using the DAF-2DA fluorophore and CLMS the •NO presence in *P. patens* protonema was confirmed and showed that •NO is distributed throughout the cytoplasm (Fig. 5). The DAF-2DA signal intensity was reduced when plants were treated with the •NO scavenger cPTIO, demonstrating that the signal corresponded specifically to •NO presence. The DAF-2DA signal intensity in plants with impaired NR activity was also diminished, confirming the observed effect of sodium tungstate on the •NO concentration obtained by EPR (Fig. 3). The results presented in Fig. 5A show a faint green signal in tungstate-treated protonema, but according to the quantitative analysis their comparative fluorescence is in the order of control plants treated with cPTIO (Fig. 5C), demonstrating that in tungstate-treated plants the •NO level was diminished.

Overall, the information available and the data presented here demonstrate that NR is the main enzymatic source of •NO synthesis in *P. patens*. The presence of •NO in protonema indicates that this molecule is needed for *P. patens* normal growth, which was expected, due to the known crucial roles of •NO in plant physiology. Our results are the first step in a promising new area of research that necessarily leads to the analysis of the probable cross-talk between •NO and other plant hormones that has been demonstrated in higher plants (Recently reviewed in [67]). In *P. patens*, auxin and cytokinins are important for differentiation of caulonema to chloronema and for caulonema branching, respectively [68]. It is possible that •NO is involved in these development processes. Furthermore, protonema growth occurs by tip growth and it was shown that •NO plays a crucial role in the control of tip growth in root hairs and pollen tube of angiosperms [69, 33]. Hence, the analysis of the roles of •NO in *P. patens* growth, development and physiology will open the door to understand the evolution of this gas molecule in plant biology.

## Supporting Information

S1 Fig Effect of high nitrate on *P. patens* protonema growth and NR activity. The effect of high nitrate in growth A) and NR activity B) was analyzed in plants that grew for seven days in Knop media alone or with 8.4 mM Ca(NO3)2, supplemented or not with 30μM sodium tungstate. Relative growth rate was measured according the formula: (*ln a*_*f*_ *—ln a*_*0*_ *)t* ^*-1*^ where *a*_*f*_ and *a*_*0*_ are the plant area at day 7 and at day 0, respectively. *t* is the time growth in days = 7. Data are mean of three independent experiments. Data were analyzed by one-way ANOVA and Tukey’s multiple comparison test (*n* = 56 in panel A and *n* = 7–9 in panel B). Different letters indicate a statistically significant difference (*P* < 0.05). Error bars denote SE.
(TIF)

## References

[1] Durner J, Klessig DF. Nitric oxide as a signal in plants. *Curr* Opin Plant Biol. 1999;2: 369–374. 10508751 10.1016/s1369-5266(99)00007-2

[2] Lamattina L, Garcia-Mata C, Graziano M, Pagnussat G. Nitric oxide: the versatility of an extensive signal molecule. Annu Rev Plant Biol. 2003;54: 109–136. 14502987 10.1146/annurev.arplant.54.031902.134752

[3] Gupta KJ, Fernie AR, Kaiser WM, Dongen JT. On the origins of nitric oxide. Trends Plant Sic. 2011;16: 160–168. 10.1016/j.tplants.2010.11.007 21185769

[4] Beligni MV, Lamattina L. Nitric oxide stimulates seed germination and de-etiolation, and inhibits hypocotyl elongation, three light-inducible responses in plants. Planta. 2000;210: 215–221. 10664127 10.1007/PL00008128

[5] Hung KT, Kao CH. Nitric oxide counteracts the senescence of rice leaves induced by abscisic acid. J Plant Physiol. 2003;160: 871–879. 12964863 10.1078/0176-1617-01118

[6] He Y, Tang RH, Hao Y, Stevens RD, Cook CW, Ahn SM, et al Nitric oxide represses the Arabidopsis floral transition. Science 2004;305: 1968–1971. 15448272 10.1126/science.1098837

[7] Delledonne M, Xia Y, Dixon RA, Lamb C. Nitric oxide functions as a signal in plant disease resistance. Nature. 1998;394: 585–588. 9707120 10.1038/29087

[8] García-Mata C, Lamattina L. Nitric oxide induces stomatal closure and enhances the adaptative plant responses against drought stress. Plant Physiol. 2001;123: 1196–1204. 10.1104/pp.126.3.1196 PMC116475 11457969

[9] Kolbert S, Ortega L, Erdei L. Involvement of nitrate reductase (NR) in osmotic stress.induced NO generation of *Arabidopsis thaliana* L. roots. *J* Plant Physiol. 2010;167: 77–80. 19822377 10.1016/j.jplph.2009.08.013

[10] Gas E, Flores-Pérez U, Sauret-Güeto S, Rodríguez-Concepción M. Hunting for plant nitric oxide synthase provides new evidence of a central role for plastids in nitric oxide metabolism. Plant Cell. 2009;21: 18–23 10.1105/tpc.108.065243 19168714 PMC2648077

[11] Palavan-Unsal N, Arisan D. Nitric oxide signalling in plants. Bot Rev. 2009;75: 203–229.

[12] Mayer B, Hemmens B. Biosynthesis and action of nitric oxide in mammalian cells. Trends Biochem Sci. 1997;22: 477–481. 9433128 10.1016/s0968-0004(97)01147-x

[13] Cueto M, Hernández-Perera O, Martín R, Bentura ML, Rodrigo J. Presence of nitric oxide synthase activity in roots and nodules of *Lupinus albus* . FEBS Lett. 1996;398: 159–64 8977098 10.1016/s0014-5793(96)01232-x

[14] Ribeiro EA, Cunha FQ, Tamashiro WM, Martins IS. Growth phase-dependent subcellular localization of nitric oxide synthase in maize cells. FEBS Lett. 1999;445: 283–86. 10094473 10.1016/s0014-5793(99)00138-6

[15] Tun NN, Holck A, Scherer GFE. Rapid increase of NO release in plant cells cultures induced by cytokinin. FEBS Lett. 2001;509: 174–176. 11741583 10.1016/s0014-5793(01)03164-7

[16] Foissner I, Wendehenne D, Langebartels C, Durner J. In vivo imaging of an elicitor-induced nitric oxide burst in tobacco. Plant J. 2000;23: 817–824. 10998192 10.1046/j.1365-313x.2000.00835.x

[17] Barroso JB., Corpas FJ, Carreras A, Sandalio LM, Valderrama R, Palma JM, et al Localization of nitric-oxide synthase in plant peroxisomes. J Biol Chem. 1999;274: 36729–36733. 10593979 10.1074/jbc.274.51.36729

[18] Pedroso MC, Magalhaes JR, Durzan D. A nitric oxide burst precedes apoptosis in angiosperm and gymnosperm callus cells and foliar tissues. J Exp Bot. 2000;51: 1027–1036. 10948230 10.1093/jexbot/51.347.1027

[19] Yu L, Wu X, Ye J, Zhang S, Wang C. NOS-like-mediated nitric oxide is involved in *Pinus thunbergii* response to the invasion of *Bursaphelenchus xylophilus* . Plant Cell Rep. 2012;31: 1813–1821. 10.1007/s00299-012-1294-0 22674219

[20] Hancock JT. NO synthase? Generation of nitric oxide in plants. Period Biol. 2013;114: 19–24.

[21] Foresi N, Correa-Aragunde N, Parisi G, Caló G, Salerno G, Lamattina L. Characterization of a nitric oxide synthase from the plant kingdom: •NO generation from the green alga *Ostreococcus tauri* is light irradiance and growth phase dependent. Plant Cell. 2010;22: 3816–3830 10.1105/tpc.109.073510 21119059 PMC3015112

[22] Dean JV, Harper JE. Nitric oxide and nitrous oxide production by soybean and winged bean during the *in vivo* nitrate reductase assay. Plant Physiol. 1986;82: 718–723. 16665099 10.1104/pp.82.3.718 PMC1056196

[23] Yamasaki H, Sakihama Y. Simultaneous production of nitric oxide and peroxynitrite by plant nitrate reductase: in vitro evidence for the NR-dependent formation of active nitrogen species. FEBS Lett. 2000;468: 89–92. 10683447 10.1016/s0014-5793(00)01203-5

[24] Rockel P, Strube F, Rockel A, Wildt J, Kaiser WM. Regulation of nitric oxide (NO) production by nitrate reductase *in vivo* and *in vitro* . J Exp Bot. 2002;53: 103–110. 11741046

[25] Meyer C, Lea US, Provan F, Kaiser WM, Lillo C. Is nitrate reductase a major player in the plant NO (nitric oxide) game? Photosynth Res. 2005;83: 181–189. 16143851 10.1007/s11120-004-3548-3

[26] Modolo LV, Augusto O, Almeida IMG, Pinto-Maglio CAF, Oliveira HC, Seligman K, et al Decreased arginine and nitrite levels in nitrate reductase-deficient *Arabidopsis thaliana* plants impair nitric oxide synthesis and the hypersensitive response to *Pseudomonas syringae* . Plant Sci. 2006;171: 34–40.

[27] Seligman K, Saviani EE, Oliveira HC, Pinto-Maglio CAF, Salgado I. Floral transition and nitric oxide emission during flower development in Arabidopsis thaliana is affected in nitrate reductase-deficient plants. Plant Cell Physiol. 2008;49: 1112–1121. 10.1093/pcp/pcn089 18540030

[28] Perchepied L, Balagué C, Riou C, Claudel-Renard C, Rivière N, Grezes-Besset B, et al Nitric oxide participates in the complex interplay of defense-related signaling pathways controlling disease resistance to *Sclerotinia sclerotiorum* in *Arabidopsis thaliana* . Mol Plant Microbe Interact. 2010;23: 846–860. 10.1094/MPMI-23-7-0846 20521948

[29] Rasul S, Dubreuil-Maurizi C, Lamotte O, Koen E, Poinssot B, Alcaraz G, et al Nitric oxide production mediates oligogalacturonide triggered immunity and resistance to *Botrytis cinerea* in *Arabidopsis thaliana* . Plant Cell Environ. 2012;35: 1483–1499. 10.1111/j.1365-3040.2012.02505.x 22394204

[30] Mallick N, Mohn FH, Rai LC, Soeder CJ. Evidence for the non-involvement of nitric oxide synthase in nitric oxide production by the green alga *Scenedesmus obliquus* . J Plant Physiol. 2000;156: 423–426.

[31] Sakihama Y, Nakamura S, Yamasaki H. Nitric oxide production mediated by nitrate reductase in the green alga *Chlamydomonas reinhardtii*: an alternative NO production pathway in photosynthetic organisms. Plant Cell Physiol. 2002;43: 290–297. 11917083 10.1093/pcp/pcf034

[32] Tischner R, Planchet E, Kaiser W. Mitochondrial electron transport as a source for nitric oxide in the unicellular green alga *Chlorella sorokiniana* . FEBS Lett. 2004;576: 151–155. 15474028 10.1016/j.febslet.2004.09.004

[33] Wang YH, Chen T, Zhang CY, Hao HQ, Liu P, Zheng MZ, et al Nitric oxide modulates the influx of extracellular Ca^2+^ and actin filament organization during cell wall construction in *Pinus bungeana* pollen tubes. New Phytol. 2009;182: 851–862. 10.1111/j.1469-8137.2009.02820.x 19646068

[34] Silveira V, Santa-Catarina C, Tun NN, Scherer GFE, Handro W, Guerra MP, et al Polyamine effects on the endogenous polyamine contents, nitric oxide release, growth and differentiation of embryogenic suspension cultures of *Araucaria angustifolia* (Bert.) O. Ktze. Plant Sci. 2006;171: 91–98. 16771985

[35] Shaw J, Renzaglia K. Phylogeny and diversification of bryophytes. Am J Bot. 2004;91: 1557–1581. 10.3732/ajb.91.10.1557 21652309

[36] Mishler BD, Oliver MJ. Putting *Physcomitrella patens* on the tree of life: the evolution and ecology of mosses In: Knight C, Perroud PF, Cove D eds. Annual Plant Reviews Volume 36: The Moss *Physcomitrella patens*. West Sussex, UK: Wiley-Blackwell, 2009 pp. 1–15.

[37] Menand B, Yi K, Jouannic S, Hoffmann L, Ryan E, et al An ancient mechanism controls the development of cells with a rooting function in land plants. Science. 2007;316: 1477–1480. 17556585 10.1126/science.1142618

[38] Pires ND, Yi K, Breuninger H, Catarino B, Menand B, et al Recruitment and remodeling of an ancient gene regulatory network during land plant evolution. Proc Natl Acad Sci USA. 2013;110: 9571–9576. 10.1073/pnas.1305457110 23690618 PMC3677440

[39] Chen YR, Su YS, Tu SL. Distinct phytochrome actions in nonvascular plants revealed by targeted inactivation of phytobilin biosynthesis. Proc Natl Acad Sci USA. 2012;109: 8310–8315. 10.1073/pnas.1201744109 22566621 PMC3361420

[40] Xu B, Ohtani M, Yamaguchi M, Toyooka K, Wakazaki M, et al Contribution of NAC transcription factors to plant adaptation to land Science. 2014;343: 1505–1508. 10.1126/science.1248417 24652936

[41] Medina-Andrés R, Lira-Ruan V. *In silico* characterization of a nitrate reductase gene family and analysis of the predicted proteins from the moss *Physcomitrella patens* . Commun Integr Biol. 2012;5:19–25. 22482004 10.4161/cib.18534 PMC3291307

[42] Nemie-Feyissa D, Królicka A, Førland N, Hansen M, Heidari B, Lillo C. Post-translational control of nitrate reductase activity responding to light and photosynthesis evolved already in the early vascular plants. J Plant Physiol. 2013;170: 662–667. 10.1016/j.jplph.2012.12.010 23395536

[43] Mur LAJ, Mandon J, Cristescu SM, Harren FJM, Prats E. Methods of nitric oxide detection in plants: A commentary. Plant Sci. 2011;181: 509–519. 10.1016/j.plantsci.2011.04.003 21893246

[44] Hoog N. Detection of nitric oxide by electron paramagnetic resonance spectroscopy. Free Rad Biol Med. 2010;49: 122–129. 10.1016/j.freeradbiomed.2010.03.009 20304044 PMC2916063

[45] Corpas FJ, Barroso JB, Carreras A, Quirós M, León AM, Romero-Puertas MC, et al Cellular and subcellular localization of endogenous nitric oxide in young and senescent pea plants. Plant Physiol. 2004;133: 2722–2733. 10.1104/pp.104.042812 PMC523336 15347796

[46] Jasid S, Simontacchi M, Bartoli CG, Puntarulo S. Chloroplasts as a nitric oxide cellular source. Effect of reactive nitrogen species on chloroplastic lipids and proteins. Plant Physiol. 2006;142: 1246–1255. 16980561 10.1104/pp.106.086918 PMC1630751

[47] Xie Y, Mao Y, Lai D, Zhang W, Zheng T, Shen W. Roles of NIA/NR/NOA1-dependent nitric oxide production and HY1 expression in the modulation of Arabidopsis salt tolerance. J Exp Bot. 2013; 64: 3045–3060. 10.1093/jxb/ert149 23744476 PMC3741688

[48] Kojima H, Nagano T. Fluorescence indicartors for nitric oxide. Adv Mater. 2000;12: 736–738.

[49] Wang JW, Wu JY. Nitric oxide is involved in methyl jasmonate-induced defense responses and secondary metabolism activities of *Taxus* cells. Plant Cell Physiol. 2005;46: 923–930. 15829512 10.1093/pcp/pci098

[50] Lin A, Wang Y, Tang J, Xue P, Li C, Liu L, et al Nitric oxide and protein S-nitrosylation are integral to hydrogen peroxide-induced leaf cell death in rice. Plant Physiol. 2012;158: 451–464. 10.1104/pp.111.184531 22106097 PMC3252116

[51] Reski R, Abel WO. Induction of budding on chloronemata and caulonemata of the moss, *Physcomitrella patens*, using isopentenyladenine. Planta. 1985;165: 354–358. 10.1007/BF00392232 24241140

[52] Chiariello NR, Mooney HA, Williams K. Growth, carbon allocation and cost of plant tissues In: Pearcy R.W. et al (Eds), Plant Physiological ecology: field methods and instrumentation. Chapman and Hall, New York, 1989 pp. 327–365.

[53] Sheible W, Lauerer M, Shulze E, Caboche M, Stitt M. Accumultion of nitrate in the shoot act as signal to regulate shoot-root allocation in tobacco. Plant J. 1997;11: 671–691.

[54] Vandelle E, Delledonne M. Methods for Nitric Oxide Detection during Plant–Pathogen Interactions. Methods Enzymol. 2008;437: 575–574. 10.1016/S0076-6879(07)37029-8 18433648

[55] Samouilov A, Zweier JL. Analytical Implications of Iron Dithiocarbamatesfor Measurement of Nitric Oxide. Methods Enzymol 2002; 352: 505–522. 10.1016/s0076-6879(02)52044-9 12125375

[56] Planchet E, Gupta KJ, Sonoda M, Kaiser WM. Nitric oxide emission from tobacco leaves and cell suspensions: rate limiting factors and evidence for the involvement of mitochondrial electron transport. Plant J. 2005;41: 732–743. 15703060 10.1111/j.1365-313X.2005.02335.x

[57] Zhao M, Chen L, Zhang L, Zhang W. Nitrate reductase-dependent nitric oxide production is involved in cold acclimation and freezing tolerance in Arabidopsis. Plant Physiol. 2009;151: 755–767. 10.1104/pp.109.140996 19710235 PMC2754647

[58] Lea US, Hoopen F, Provan F, Kaiser WM, Meyer C, Lillo C. Mutation of the regulatory phosphorylation site of tobacco nitrate reductase results in high nitrite excretion and NO emission from leaf and root tissue. Planta. 2004;219: 59–65. 14767769 10.1007/s00425-004-1209-6

[59] Hayat S, Yadav S, Wani AS, Irfan M, Alyemini MN, Ahmad A. Impact of sodium nitroprusside on nitrate reductase, proline content, and antioxidant system in tomato under salinity stress. Hortic Environ Biotech. 2012;56: 362–367.

[60] Besson-Bard A, Astier J, Rasul S, Wawer I, Dubreuil-Maurizi C, Jeandroz S, et al Current view of nitric oxide-responsive genes in plants. Plant Sci. 2009;177: 302–309.

[61] Lehner C, Kerschbaum HH, Luz-Meindl U. Nitric oxide suppresses growth and development in the unicellular green alga *Micrasterias denticulata* . J Plant Physiol. 2009;166: 117–127. 10.1016/j.jplph.2008.02.012 18455833

[62] Xiong J, Fu G, Yang Y, Zhu C, Tao L. Tungstate: is it really a specific nitrate reductase inhibitor in plant nitric oxide research? J Exp Bot. 2012;63: 33–41. 10.1093/jxb/err268 21914661

[63] Millar TM, Stevens CR, Benjamin N, Eisenthal R, Harrison R, et al Xanthine oxidoreductase catalyses the reduction of nitrates and nitrite to nitric oxide under hypoxic conditions. FEBS Lett. 1998;427: 225–228. 9607316 10.1016/s0014-5793(98)00430-x

[64] Doel JJ, Godber BLJ, Eisenthal R, Harrison R. Reduction of organic nitrates catalysed by xanthine oxidoreductase under anaerobic conditions. Biochi Biophys Acta. 2001;1527: 81–87. 11420146 10.1016/s0304-4165(01)00148-9

[65] Wilkinson JQ, Crawford NM. Identification and characterization of a chlorate resistant mutant of *Arabidopsis thaliana* with mutations in both nitrate reductase structural genes NIA1 and NIA 2. Mol Gene Genetics. 1993;239: 289–297. 8510658 10.1007/BF00281630

[66] Abu-Abied M, Szwerdszarf D, Mordehaev I, Levy A, Rogovoy O, Belausov E, et al Microarray analysis revealed upregulation of nitrate reductase in juvenile cuttings of *Eucalyptus grandis*, which correlated with increased nitric oxide production and adventitious root formation. Plant J. 2012;71: 787–799 10.1111/j.1365-313X.2012.05032.x 22519851

[67] Simontacchi M, García-Mata C, Bartoli CG, Santa-María GE, Lamattina L. Nitric oxide as a key component in hormone-regulated processes. Plant Cell Rep. 2013;32: 853–866. 10.1007/s00299-013-1434-1 23584547

[68] Decker EL, Frank W, Sarnighausen E, Reski R. Moss systems biology en route: phytohormones in *Physcomitrella* development. Plant Biol. 2006;8: 397–405. 16807833 10.1055/s-2006-923952

[69] Lombardo MC, Graziano M, Polacco JC, Lamattina L. Nitric oxide functions as a positive regulator of root hair development. Plant Signal Behav. 2006;1: 28–33. 19521473 10.4161/psb.1.1.2398 PMC2633697

