# Supplementary figures and images for: The Nitric Oxide Production in the Moss Physcomitrella patens Is Mediated by Nitrate Reductase
Source: PLoS One. 2015 Mar 5;10(3):e0119400. doi: 10.1371/journal.pone.0119400 (PMC4351199; doi:10.1371/journal.pone.0119400)

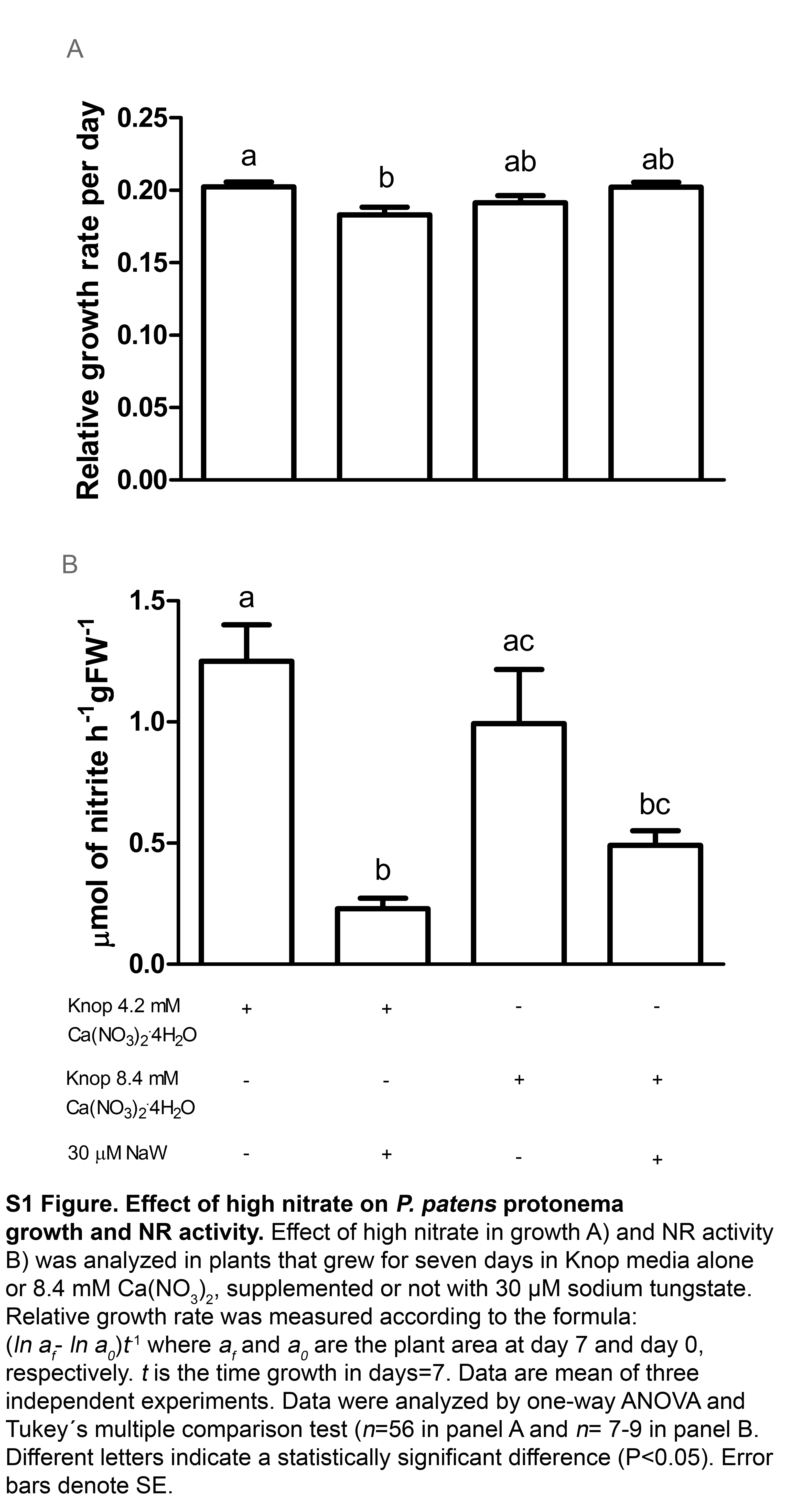

Supplement: S1 Fig — The effect of high nitrate in growth A) and NR activity B) was analyzed in plants that grew for seven days in Knop media alone or with 8.4 mM Ca(NO3)2, supplemented or not with 30μM sodium tungstate. Relative growth rate was measured according the formula: (ln a f —ln a 0 )t -1 where a f and a 0 are the plant area at day 7 and at day 0, respectively. t is the time growth in days = 7. Data are mean of three independent experiments. Data were analyzed by one-way ANOVA and Tukey’s multiple comparison test (n = 56 in panel A and n = 7–9 in panel B). Different letters indicate a statistically significant difference (P < 0.05). Error bars denote SE. (TIF) [file pone.0119400.s001.tif]
